# Case Report: Reconciling genomic ambiguity with clinical certainty in refractory panniculitis

**DOI:** 10.3389/fimmu.2026.1906895

**Published:** 2026-09-15

**Authors:** Sayan Mukherjee, Rajat K. Sahu, Nidhish M. Chandra, Urmila Dhakad, Puneet Kumar

**Affiliations:** 1 Department of Clinical Immunology and Rheumatology, Rabindranath Tagore International Institute of Cardiac Sciences (RTIICS), Kolkata, India; 2 Department of Clinical Immunology and Rheumatology, King George’s Medical University, Lucknow, India

**Keywords:** adolescent health, autoinflammatory and autoimmune diseases, HAVCR2, HLH - hemophagocytic lymphohistiocytosis, novel variant, panniculitis, refractory panniculitis, skin inflammation

## Abstract

Panniculitis in adolescents often poses a diagnostic challenge for physicians. We report an adolescent girl presenting with recurrent steroid-responsive lymphocytic septal panniculitis, genital ulcer, and constitutional symptoms, later complicated by macrophage activation syndrome and steatohepatitis. Extensive evaluation excluded infectious, autoimmune, and malignant causes. Histopathology revealed septal panniculitis without vasculitis or atypical lymphoid cells. Whole-exome sequencing identified a heterozygous *PSMB9* variant (c.595A>G; p.Thr199Ala) and a homozygous *HAVCR2* variant (c.290T>C; p.Ile97Thr); the latter has not been reported in a homozygous state in existing genomic databases and is predicted to be deleterious. The coexistence of panniculitis, haemophagocytic lymphohistiocytosis (HLH)-like manifestations, and histologically lymphocytic rather than neutrophilic infiltrates suggests a distinct panniculitis–HLH overlap syndrome associated with the *HAVCR2* mutation, which does not fit within the subcutaneous panniculitis-like T-cell lymphoma spectrum. A potential contribution (gene dosage effect) from the heterozygous *PSMB9* mutation was uncertain, as parental testing was not feasible. The patient achieved sustained remission on corticosteroids and tacrolimus. This case broadens the phenotypic spectrum of *HAVCR2*-associated immune dysregulation and documents a novel homozygous *HAVCR2* variant (p.Ile97Thr), underscoring the importance of comprehensive genetic evaluation in patients with unexplained recurrent panniculitis.

## Introduction

Panniculitis refers to inflammation of the subcutaneous adipose tissue and can be histologically classified into septal or lobular types based on the predominant site of involvement. The aetiology of panniculitis varies across age groups, and a broad spectrum of diseases can present with recurrent episodes. Accurate diagnosis relies on histopathological evaluation, taking into account the pattern of involvement, the predominant type of inflammatory cell infiltrate, and the presence or absence of associated vasculitis. In children and adolescents, panniculitis may result from infections, connective tissue diseases, or autoinflammatory disorders, with or without underlying immunodeficiencies (1, 2).

Erythema nodosum (EN) is the most common form of panniculitis across all age groups, with a peak incidence during adolescence and no clear sex predilection (3). In children, lesions typically involve the anterior thighs, upper limbs, trunk, and face. Streptococcal and gastrointestinal infections are the most frequently identified triggers, though up to 40% of paediatric cases remain idiopathic. Histologically, EN in children resembles the adult form, displaying a predominantly septal panniculitis. The inflammatory infiltrate varies with lesion age: acute lesions are neutrophil-rich, while chronic lesions show a predominance of mononuclear cells and histiocytes. Miescher’s radial granulomas—stellate aggregates of histiocytes—may aid diagnosis but are not consistently present (1).

We present a diagnostically challenging case of refractory panniculitis complicated by haemophagocytic lymphohistiocytosis (HLH), in which genomic interrogation revealed a novel variant of uncertain significance. Although the genetic findings did not meet established criteria for pathogenicity, meticulous clinical phenotyping and a mechanistic understanding of the underlying immune dysregulation facilitated syndrome recognition and therapeutic intervention. The resultant sustained remission underscores the critical importance of integrating genomic data with clinical and biological context, particularly when variant interpretation remains inconclusive. This case highlights the evolving paradigm of precision immunology, where pathogenic inference and therapeutic decision-making are guided not solely by genotype but by the convergence of molecular, clinical, and immunological evidence.

## Case presentation

A 14-year-old girl presented with a 1-year history of intermittent high-grade fever, 10 months of recurrent painful erythematous cutaneous nodules, and a 6-month history of a painful solitary vulval ulcer. The fever was insidious and intermittent, lacked diurnal variation, and responded poorly to antipyretics. Cutaneous lesions began as well-circumscribed, tender nodules over the buttocks, spreading to the trunk within 1 month and subsequently to the extremities. Lesions healed with hyperpigmentation without ulceration and spared the face. Over the preceding 8 months, some gluteal lesions developed superficial ulceration. She was initially treated for a gluteal abscess with multiple antibiotic courses without improvement. Empirical antitubercular therapy was initiated for suspected erythema nodosum but discontinued due to transaminitis. Concurrently, a vulval papule progressed to a deep ulcer with undermined edges. A thigh lesion subjected to incision and drainage subsequently ulcerated and failed to heal.

5 months prior, she was evaluated at our center and diagnosed with undifferentiated vasculitis. Treatment with prednisolone (0.5 mg/kg/day) and methotrexate was initiated; however, methotrexate was discontinued due to recurrent transaminitis and replaced with colchicine. Following steroid tapering to < 10 mg/day, she developed a relapse, necessitating dose escalation and the addition of azathioprine.

She then presented with a 15-day history of persistent fever and recurrence of nodular skin lesions over the trunk and lower limbs, accompanied by severe fatigue and diffuse arthralgia. She reported adherence to prednisolone (5 mg/day) and azathioprine (100 mg/day), with no new drug exposures. **Figure 1** summarizes the timeline of clinical events.

**Figure 1:**
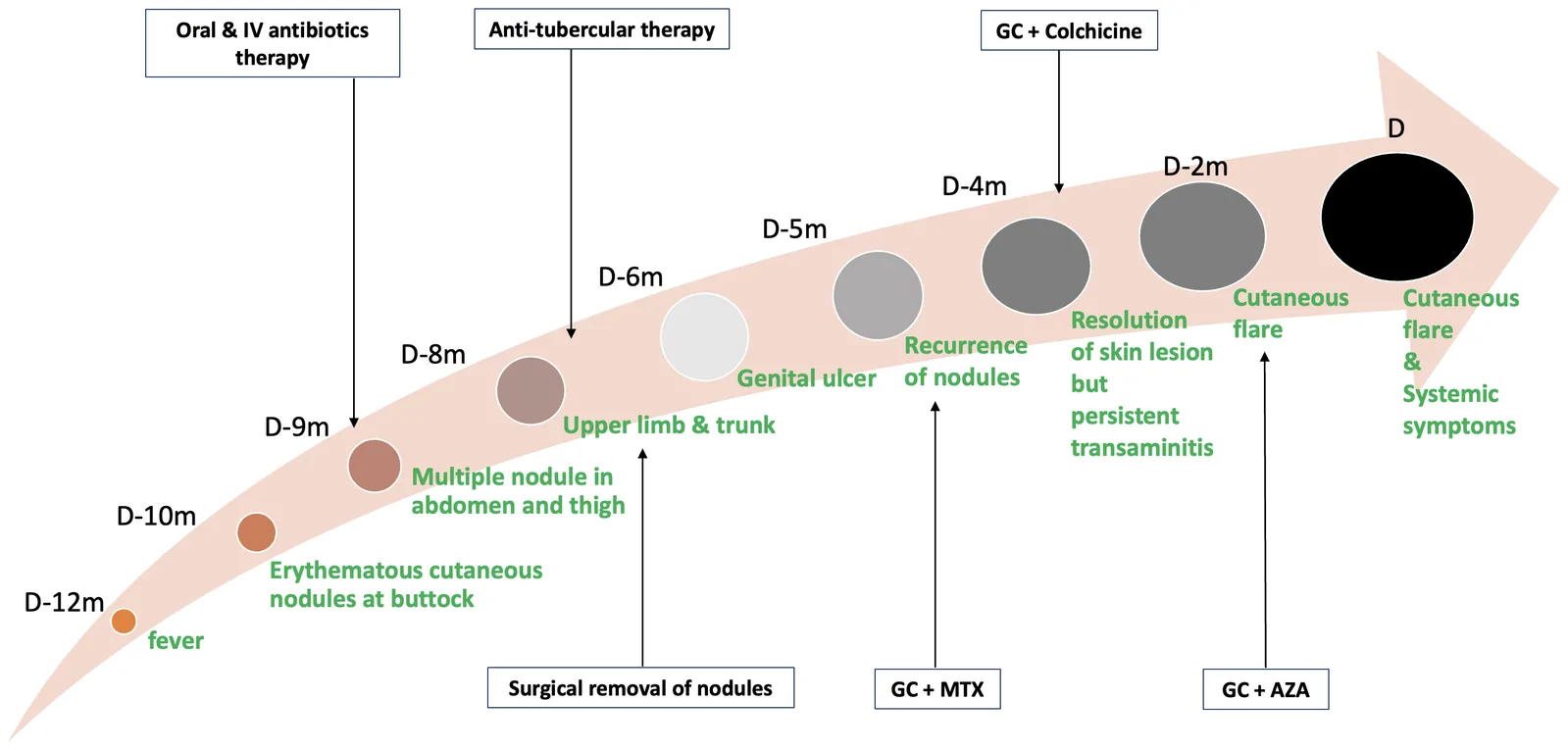
Timeline of events. D, present day; m, months; D-12 m, episode 12 months previously; and the remaining time points are similarly defined. GC, glucocorticoid; MTX, methotrexate; AZA, azathioprine.

There was no significant past medical or surgical history apart from vasculitis, and no family history of tuberculosis or autoimmune disease. No known genetic conditions or consanguinity were recorded in the family. She had normal developmental milestones, had not yet attained menarche, and had experienced anorexia, fatigue, and a 5 kg weight loss over 6 months. She had previously performed well academically and was fully immunized as per national guidelines. A comprehensive review of systems was otherwise unremarkable.

On examination, the patient appeared to be of average build but poorly nourished, with a body mass index (BMI) of 16 kg/m². She was pale and febrile (102°F) but haemodynamically stable. Cutaneous examination revealed multiple erythematous, tender, subcutaneous nodules measuring 1–2 cm in diameter, distributed over the abdomen, buttocks, thighs, legs, and upper limbs. The lesions were in various stages of healing; several showed induration and hyperpigmentation. A single, well-defined, erythematous, tender vulval ulcer with an undermined edge and serous discharge was present. Otherwise, her systemic examination was unremarkable.

Serial laboratory parameters (**Table 1**) demonstrated iron-deficiency anemia, transaminitis, markedly elevated alkaline phosphatase, and raised inflammatory markers at the initial presentation. Hepatic dysfunction was initially attributed to possible granulomatous hepatitis in the context of suspected tuberculosis, supported by clinical features; abdominal ultrasound showed mild hepatomegaly without parenchymal disease. Liver enzymes further worsened following the initiation of antitubercular therapy. Subsequent evaluation led to a working diagnosis of vasculitis, and she was treated with glucocorticoids and methotrexate. Skin biopsy revealed septal panniculitis without vasculitis, and immunohistochemistry demonstrated a mixed CD4/CD8 T-cell infiltrate without clonality (**Figure 2**). Methotrexate was later discontinued due to suspected drug-induced transaminitis and replaced with colchicine. Although anemia partially improved, a disease flare necessitated the initiation of azathioprine.

**Table 1:** Serial laboratory tests and medical records of the patient.

Serial laboratory tests and medical records of the patient											
Therapeutic intervention offered					Methotrexate		Colchicine		Azathioprine		TAC
					Glucocorticoids						
			ATT							Pulse MP x3d +2unit PRBC	
	IV antibiotics										
Timeframe	D-10m	D-9m	D-7m	D-6m	D-6m	D-5m	D-4m	D-2m	D-1m	D	D+15
Hb	8.8	9.0	9.8	9.2	–	10.2	11.8	11.5	11.8	6.9	9.8
TLC	6000	5900	6100	5791	–	18200	8600	6800	6000	2870	4600
PLT	3.3	3.5	4.6	3.2	–	3.04	1.56	1.67	1.08	0.77	1.32
N%/L%	70/25	72/25	–	–	–	–	73/12	69/25	80/16	90/06	88/10
MCV	81	82	–	–	–	–	84	–	79	88	80
ESR	–	60	–	22	–	9	32	–	22	10	19
CRP	–	30.8	26	16.28	–	8.6	5.6	12.2	–	25	8
Total Bilirubin	0.27	0.63	1.28	1.27	–			–	–	1.9	–
Direct Bilirubin	0.17	0.24	0.9	0.94	–			–	–	0.8	–
SGPT	104	97	131	230	86	192	23	89	42	105	58
SGOT	240	240	246	470	92	221	88	56	37	82	46
ALP	3433	2861	3715	2623	1782	692	372	164	–	246	–
Creatinine	0.4	0.5	0.6	0.5	–	0.52	0.73	0.55	–	0.8	0.7
Urea	18	20	–	–	–	–	29	24	–	22	–
Total protein	–	–	–	5.6	–	5.7	–	–	–	6.4	–
Albumin	–	–	–	2.3	–	3.4	–	–	–	3.2	–
Ferritin	26	–	–	–	–	–	–	–	–	1008	582
Fibrinogen	–	–	–	–	–	–	–	–	–	280	–
Triglyceride	–	–	–	–	–	–	–	–	–	390	–
Procal.	–	–	–	–	–	–	–	–	–	0.09	0.04
Autoimmune profile • ANA by IIF (1:100): Negative • ENA: Negative • MSA (IP): Negative • AntidsDNA-Elisa: <20 • C3:176 • C4: 96.2 • Anti-LKM-1: Negative • Anti-SLA: Negative • AMA: Negative • Anti-PR3: Negative • Anti-MPO: Negative • Fecal calprotectin: <45μg/mg			Infection profile • HbsAg/ Anti-HCV: Non-reactive • Anti-HAV IgM/ Anti-HEV IgM: Non-reactive • HIV1&2: Non-reactive • CMV/EBV/Parvo B19 PCR- Negative • Urine R&M: NAD • Urine culture: No growth • Blood c/s: No growth • Serial wound swab c/s: No growth • Vulval swab c/s: Pseudomonas sp. • Mantoux test : 2mm x 4mm (Negative)				Imaging • US abdomen and pelvis: NAD • Chest X ray: NAD • Contrast enhanced CT chest and abdomen: hepatomegaly				
Biopsy reports Skin biopsy: Septal panniculitis without any granuloma or vasculitis *(**Figure 2**)* Final Dx: Erythema nodosum Liver biopsy: Hepatocytes show micro and macro vesicular steatosis with mild peri-portal lymphocytic inflammation with preserved parenchymal architecture. No evidence of rosette or necroinflammation or fibrosis. Final Dx: Steatohepatitis Bone marrow biopsy: Haematopoiesis with evidences of Hemophagocytosis and histiocytosis. Final Dx: Hemophagocytosis											

HB, Hemoglobin(g/dl); TLC, total leukocyte count(per mm^3^); PLT, platelet count(X10^9^ per mm^3^); N%/L%, neutrophil & Lymphocyte(%); MCV, Mean corpuscular volume(fl); ESR, Erythrocyte sedimentation rate(mm/1^st^ hour); CRP, C reactive protein(mg/L); SGPT, Serum Glutamic-Pyruvic Transaminase(IU/L); SGOT, Serum Glutamic-Oxaloacetic Transaminase(IU/L); ALP, Alkaline phosphatase(U/L); Procal, Procalcitonin(ug/L). Expression units: Ferritin (ug/L), triglyceride(mg/dl), creatinine(mg/dl), fibrinogen(mg/dl) and protein & albumin (g/dl).

TAC, Tacrolimus; ANA, anti-nuclear antigen; ENA, extractable nuclear antigen; MSA, Myositis specific antibody; dsDNA, double stranded deoxyribonucleic acid; C3, complement protein3; C4, complement protein4; Anti-LKM-1, Liver kidney microsome type1 antibody; SLA, Soluble liver antigen; AMA, anti-mitochondrial antibody; IIF, indirect immunofluorescence; IP, Immunoprecipitation; Elisa, Enzyme linked immunosorbent assay; HbsAg, Hepatitis B surface antigen; HCV, Hepatitis C virus; HAV, Hepatitis A virus; HEV, Hepatitis E virus; HIV, Human immunodeficiency virus; CMV, Cytomegalo virus; EBV, Epstein Barr virus; NAD, No abnormality detected.

**Figure 2:**
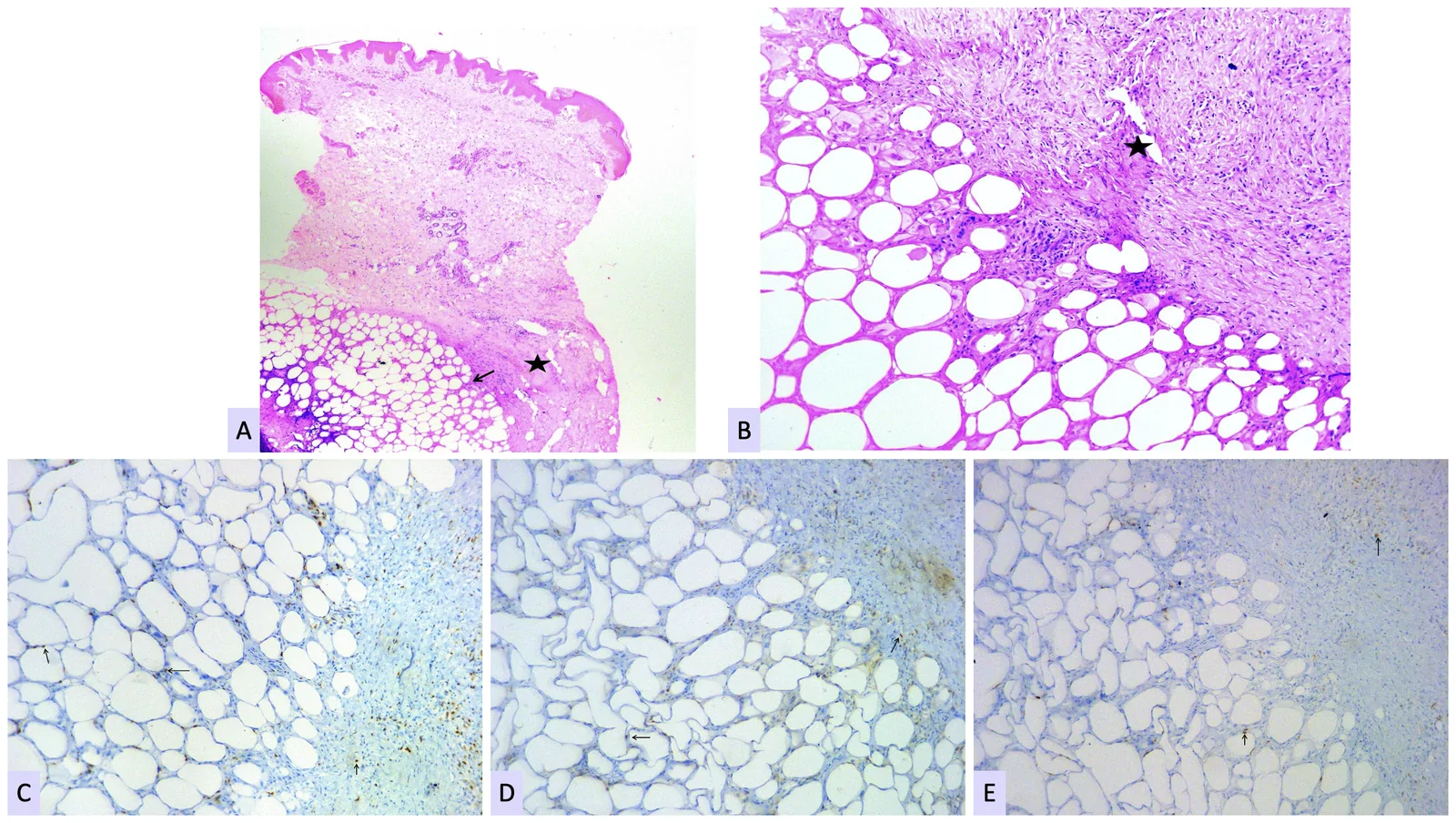
**(A)** Section shows skin with subcutaneous septal fibrosis and chronic inflammation (asterisk), extending into surrounding lobular fat (arrow). Haematoxylin and eosin, ×25; **(B)** section shows subcutaneous fat with septal fibrosis and a septal infiltrate of lymphocytes, histiocytes, and few eosinophils (asterisk). Haematoxylin and eosin, ×200; **(C)** section shows subcutaneous fat with a scattered infiltrate of CD3-positive T lymphocytes (arrows). Adipocytic rimming is not seen. Diaminobenzidine, ×100; **(D)** section shows subcutaneous fat with a scattered infiltrate of CD4-positive T lymphocytes (arrows). Diaminobenzidine, ×100; **(E)** section shows subcutaneous fat with a scattered infiltrate of CD8-positive T lymphocytes (arrows). Diaminobenzidine, ×100.

At this presentation, she developed acute pancytopenia, transaminitis, hyperferritinaemia, and hypertriglyceridaemia, consistent with macrophage activation syndrome in the setting of a disease flare. Infectious triggers were excluded, and imaging showed no evidence of occult infection or malignancy. Bone marrow examination confirmed haemophagocytosis, while liver biopsy demonstrated non-specific steatosis. She was managed with a packed red cell transfusion and intensive immunosuppression, resulting in clinical and biochemical improvement over 2 weeks.

## Case summary

An adolescent girl presented with steroid-responsive recurrent septal panniculitis (erythema nodosum), a genital ulcer, and constitutional symptoms and was admitted with a cutaneous flare, haemophagocytosis, and steatohepatitis.

### Differential diagnosis

A broad differential diagnosis was considered for recurrent septal panniculitis with systemic manifestations. Behçet’s disease was initially considered in view of erythema nodosum-like lesions and genital ulcers; however, the absence of oral aphthae, negative pathergy, modest inflammatory markers, and lack of vasculitis on histopathology, together with peripheral haemophagocytosis, argued against this diagnosis. Systemic lupus erythematosus was deemed unlikely given the negative serology and the presence of septal rather than lobular panniculitis. Subcutaneous panniculitis-like T-cell lymphoma remains a diagnostic consideration; however, the relapsing course, steroid responsiveness, and absence of classical peri-adipocyte rimming are atypical for this entity. Monogenic autoinflammatory disorders, including haploinsufficiency of A20 (HA20), were considered less likely due to the absence of mucocutaneous, gastrointestinal, and ocular involvement and the later disease onset. Other autoinflammatory syndromes (RELopathies—ELF4/*REL*) which involve nuclear factor kappa B (NF-κB) signalling and cell death pathway (tumor necrosis factor (TNF) receptor pathway—*OTULIN*/HA20) dysregulation were also deemed less significant due to lack of a neutrophilic driver, a classical disease phenotype, and an association with HLH. Inflammatory bowel disease and sarcoidosis were effectively excluded based on unremarkable imaging and the absence of granulomatous inflammation. Proteasome-associated autoinflammatory syndromes remained within the differential but are not typically associated with haemophagocytosis or steatohepatitis. Overall, idiopathic panniculitis was considered less plausible in the context of prominent systemic and mucosal involvement.

### The patient’s course

The patient presented with a flare of panniculitis complicated by HLH-like features and was promptly started on high-dose corticosteroids. Oral tacrolimus was introduced 2 weeks later as a steroid-sparing agent, resulting in rapid clinical improvement and progressive normalization of laboratory parameters.

In view of recurrent, idiopathic panniculitis in an adolescent, whole*-*exome sequencing was undertaken. This identified a heterozygous variant in *PSMB9* (exon 6) and a homozygous variant in *HAVCR2* (exon 2), both classified as variants of uncertain significance (**Figure 3A**). While *PSMB9* mutations are implicated in proteasome-associated autoinflammatory syndromes, which may present with panniculitis, they are not typically associated with HLH-like manifestations. In contrast, *HAVCR2* variants have been reported in subcutaneous panniculitis-like T-cell lymphoma and are more strongly linked to HLH-like presentations. The precise driver of HLH in this case remains uncertain; an unrecognized or unidentified viral trigger could not be excluded, and a potential gene dosage effect of the *HAVCR2* variant was also considered.

**Figure 3:**
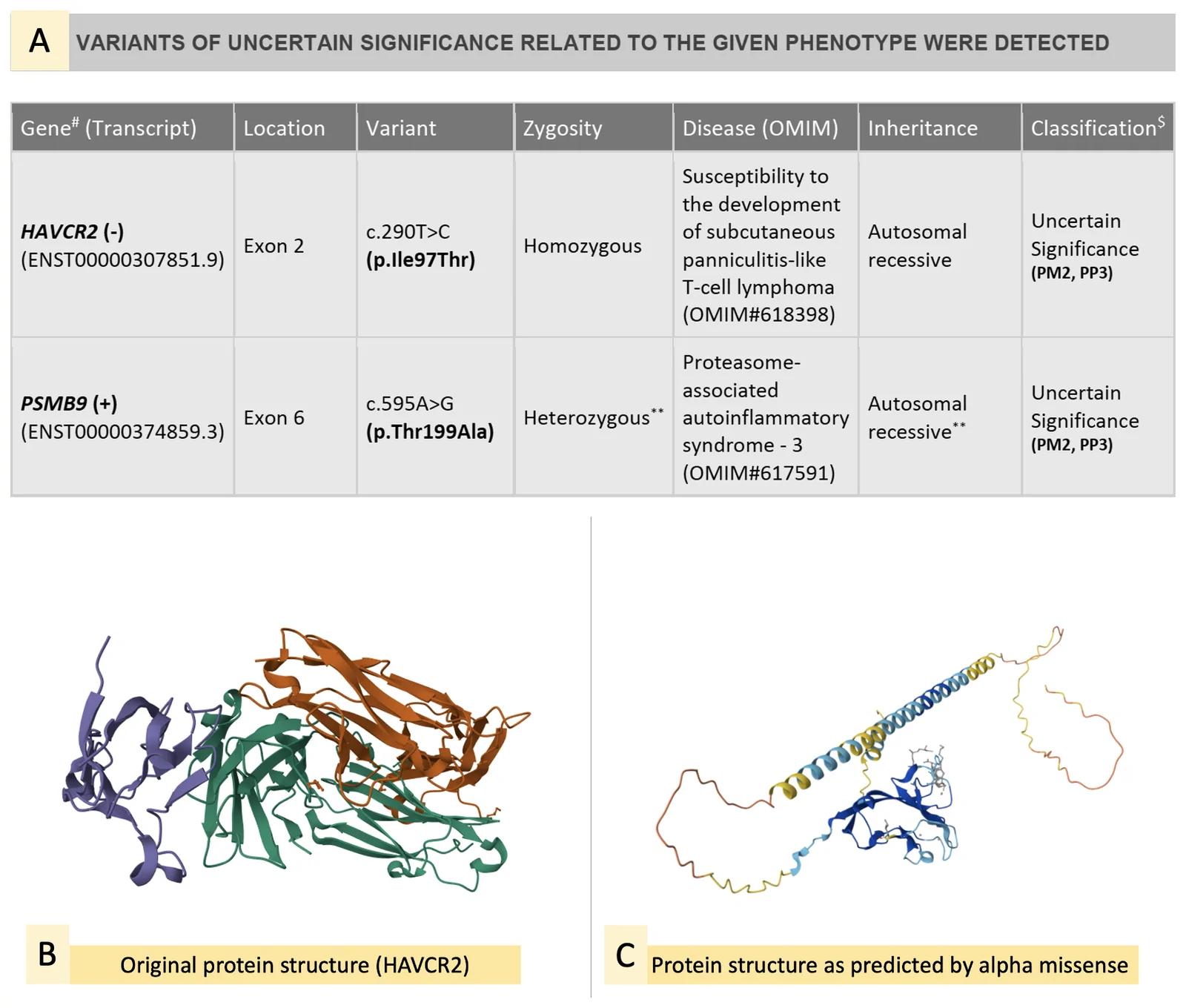
**(A)** whole-exome sequencing of the index patient with two identifiable variants of uncertain significance. **(B, C)** normal and altered protein structures associated with the *HAVCR2* index mutation, respectively.

Persistent transaminitis prompted a liver biopsy, which demonstrated non-specific steatohepatitis that was likely multifactorial—associated with prolonged corticosteroid and immunosuppressive exposure and/or systemic inflammatory activation rather than HLH alone. Although the acute dysfunction was predominantly attributable to HLH, its persistence indicated steatosis possibly secondary to medication use.

The patient was discharged on tapering corticosteroids with adjunctive tacrolimus. Over 6 months, prednisolone was successfully reduced to < 5 mg/day, while tacrolimus was maintained at 3 mg/day in divided doses, guided by therapeutic trough levels (5–8 ng/mL). She has remained in sustained clinical remission for 24 months, including over 12 months without corticosteroids, with no recurrence or disease flare.

## Discussion

In our case, the patient presented with recurrent episodes of panniculitis and fever, and was found to have a heterozygous *PSMB9* variant and a homozygous *HAVCR2* variant, in the absence of any overt or classical features of immunodeficiency. Parental genetic analysis may have provided valuable insights, but it was not performed due to financial constraints.

### Genomic analysis

The genetic workflow identified a novel, homozygous missense variant in exon 2 of the *HAVCR2* gene (chr5:g.157106731A>G; NM_032782.5: c.290T>C, p.Ile97Thr) at an analytical sequencing depth of 121×. The variant resides within the highly conserved immunoglobulin-like variable (IgV) domain of the T-cell immunoglobulin and mucin domain-containing protein 3 (TIM-3). Notably, this residue occurs at the same structural locus as the well-characterized European founder mutation p.Ile97Met, which is known to disrupt TIM-3 protein stability and drive autosomal recessive subcutaneous panniculitis-like T-cell lymphoma (SPTCL).

### Population genetics and rarity

Evaluation against the Genome Aggregation Database (gnomAD v4.1) demonstrated that the p.Ile97Thr variant is exceptionally rare in global control cohorts. The highest subpopulation minor allele frequency (MAF) was observed in the South Asian population at a negligible 0.00008783% (approximately 1 in 1.1 million alleles), followed by the non-Finnish European population at 0.000005932%. Crucially, the p.Ile97Thr mutation has never been documented in a homozygous state within gnomAD or ClinVar. To our knowledge, this index patient represents the first report in the clinical literature of this variant in a homozygous state, strongly supporting its role as a penetrant recessive disease allele.

### In silico and thermodynamic protein modelling

Computational and *in silico* meta-predictors uniformly signal high pathogenicity:

- Combined Annotation Dependent Depletion (CADD): the variant yielded a Phred score of 24.6, placing it within the top 0.5% of the most deleterious alterations in the human genome.
- Ensemble predictors: pathogenicity was strongly corroborated by Sorting Intolerant From Tolerant (SIFT) and Polymorphism Phenotyping v2 (PolyPhen-2), both of which characterized the substitution as highly damaging.
- Deep-learning classifiers: the structural impact was substantiated by AlphaMissense (score: 0.81, “Likely Pathogenic”) and Evolutionary Scale Modeling model 1b (ESM-1b; score: -12.3), indicating a severe breach of evolutionary and geometric constraints.

Biochemically, the alteration substitutes a non-polar, hydrophobic isoleucine residue with a polar, hydrophilic threonine (p.Ile97Thr). Thermodynamic modelling predicts that introducing this polar hydroxyl group into the hydrophobic core of the IgV domain causes catastrophic structural disruption, resulting in a protein destabilization free-energy change (ΔΔG) of 3.83 kcal/mol. This high-energy barrier indicates a structural collapse likely triggering protein misfolding, destabilization, and subsequent proteasomal degradation (**Figures 3B, C**).

### Reconsideration of ACMG/AMP pathogenicity classification

While currently catalogued as a variant of uncertain significance (VUS) in generic databases, a stringent synthesis of the clinical and molecular data according to the American College of Medical Genetics and Genomics/Association for Molecular Pathology (ACMG/AMP) guidelines—augmented by updated Clinical Genome Resource (ClinGen) Sequence Variant Interpretation (SVI) recommendations—reclassifies this variant as likely pathogenic via the following combination of criteria (4, 5):

- PM2 (Supporting): extreme rarity in large control cohorts (gnomAD MAF disease-specific thresholds) alongside a total absence of homozygotes.
- PM3 (Supporting): identified in a confirmed homozygous state in a patient presenting with an autosomal recessive phenotype.
- PP3: highly concordant, multi-algorithmic *in silico* and deep-learning computational evidence predicting a severe structural and evolutionary defect.
- PP4: the patient’s clinical presentation matches the highly specific, distinctive phenotypic spectrum of biallelic *HAVCR2* deficiency (SPTCL/HLH syndrome).

In conclusion, combining one Moderate and three Supporting criteria (PM2_Supporting + PM3_Supporting + PP3 + PP4) establishes a classification of likely pathogenic.

### Genomic analysis of the other variant

While *PSMB9* mutations are typically described in the context of proteasome-associated autoinflammatory syndrome with immunodeficiency (PRAAS-ID) in early infancy, they have also been implicated in other proteasome-associated autoinflammatory syndromes (PRAAS), such as PRAAS-6, PRAAS-3, and chronic atypical neutrophilic dermatosis with lipodystrophy and elevated temperature (CANDLE). However, establishing a causal link with this genetic variant remains challenging, as the variant reported in our case is a heterozygous missense mutation in the *PSMB9* gene (chr6:g.32859467A>G; depth: 135×, exon 6, c.595A>G, p.Thr199Ala) that results in the amino acid substitution of alanine for threonine at codon 199. This is a novel variant and has not been reported in the 1000 Genomes, gnomAD (v4.1), gnomAD (v2), Trans-Omics for Precision Medicine (TOPMed), or ClinVar databases, and *in silico* predictions by SIFT and the likelihood ratio test (LRT) indicate that the variant is damaging. In *PSMB9*, G156D variant is only pathogenic variant reported including digenic model. A missense mutation at a similar position (p.Thr199Ile) has been reported in the database, with an overall minor allele frequency of 0.000002479% and a CADD score of 25.8. The genomic region showed evidence of functional constraint with a genomic constraint Z of 2.77 for surrounding 1kb region. However, the reported *PSMB9* variant, p.Thr199Ala, appears to be novel and is located in a genomic region under significant negative selection. In the present context, lymphocytic rather than neutrophilic panniculitis in the active lesion argued against the diagnosis.

### Phenotypic analysis and literature review

Subcutaneous panniculitis-like T-cell lymphoma (SPTCL) is a distinct clinical entity which shows clonal lymphocytic proliferation. Clinically, it presents with erythematous, painless nodules or plaques on the limbs and trunk and, rarely, on the face, with or without accompanying systemic symptoms. It follows a relapsing and remitting course with lesions at different stages of healing (6, 7). Typical histology reveals lobular panniculitis with classical peri-adipocyte rimming of clonally proliferating T cells. Infiltrating cells are mostly cytotoxic CD8+ cells expressing the cytotoxic proteins T-cell intracellular antigen 1 (TIA1), granzyme B, and perforin, but not CD56. Immunohistochemical analysis confirms the lymphocyte clone with specific T-cell receptor (TCR) gene rearrangements (αβ or γδ) (8). The αβ variant presents at a younger age (median age 35 years) than the γδ variant. An association with HLH is reported in 15%–20% of αβ variants and in 50% of γδ variants. SPTCL is described as a CD8+ cytotoxic T-cell lymphoma expressing αβ T-cell receptors and CD4-, CD8+, and CD56- phenotypes. It is restricted to subcutaneous fat and typically has an indolent clinical course and is known as subcutaneous panniculitis-like T-cell lymphoma with an αβ T-cell phenotype (SPTL-AB). Cutaneous γδ T-cell lymphoma (CGD-TCL), also known as subcutaneous panniculitis-like T-cell lymphoma with a γδ T-cell phenotype (SPTL-GD), is characterized by a unique phenotype (CD4-, CD8-, CD56+) and has a poor prognosis. Prognosis is usually excellent in young adults but may vary in patients with an *HAVCR2* mutation (encoding TIM-3) and an HLH-like presentation (9). SPTCL patients have an excellent prognosis, with 5-year overall survival (OS) > 80%; approximately 20% develop haemophagocytic lymphohistiocytosis (HLH), which significantly reduces patient survival (5-year OS of 46%) (8). Management typically involves high-dose corticosteroids, immunotherapy, and cyclosporine.

Although *HAVCR2* is the hallmark mutation of SPTCL, varied mutational profiles have been identified across different ethnicities. The *HAVCR2 gene has three different variants—*Y82C, T101I, and I97M. The first two have been reported in individuals of Asian and Polynesian ancestry, while I97M has been found most frequently in patients with a North African or Caucasian background.

Patients with *HAVCR2* mutations have a younger median age at disease onset, a male predominance, a longer median time to diagnosis, a more severe disease course, a higher rate of autoantibodies, and a higher likelihood of systemic illness and HLH development. Two other important mutations noted in SPTCL are in the *DNMT3A* and *TP53* (P72R) genes, which are important regulators of DNA enzyme machinery and CD4 T-cell differentiation through the T helper 17/regulatory T-cell (Th17/Treg) pathway, respectively (10).

In a Korean study, *HAVCR2 ^Y82C^* was associated with younger age, the development of HLH-like illness, and shorter relapse-free survival. Most mutated genes in SPTCLs were involved in immune responses, epigenetic modifications, and cell signalling (11).

A plausible explanation for our case is that the patient’s drug-modified status may have masked the typical histological features of SPTCL. However, the clinical presentation and genotype findings are consistent with a diagnosis resembling recurrent lymphocytic panniculitis with HLH in the context of the *HAVCR2* variant. This represents a novel genetic variant with such clinical manifestations, and further studies are warranted to confirm these observations.

### Final diagnosis

Recurrent lymphocytic septal panniculitis, fever, genital ulcer, and macrophage activation syndrome with a novel homozygous *HAVCR2* variant (p.Ile97Thr) and non-specific steatohepatitis, likely multifactorial (steroid-induced rather than due to HLH alone).

### Patient’s perspective (parent’s perspective)

For several years, our daughter suffered from recurrent painful skin swellings, fever, and repeated hospital admissions. She underwent many tests and biopsies, but the cause of her illness remained unclear. As parents, it was very difficult to see her go through so much pain and uncertainty. At one stage, her condition became very serious, and we were deeply concerned about her recovery. After a long and challenging journey, the diagnosis was finally established and appropriate treatment was started. Since then, her health has improved significantly, and she has remained in remission for years. She is now able to attend school, participate in normal activities, and enjoy a much better quality of life. We are thankful to all the healthcare professionals involved in her care and for their continued support throughout this journey.
